# Screen Time and Developmental Performance Among Children at 1-3 Years of Age in the Japan Environment and Children’s Study

**DOI:** 10.1001/jamapediatrics.2023.3643

**Published:** 2023-09-18

**Authors:** Midori Yamamoto, Hidetoshi Mezawa, Kenichi Sakurai, Chisato Mori

**Affiliations:** 1 Department of Sustainable Health Science, Center for Preventive Medical Sciences, Chiba University, Chiba, Japan; 2 Medical Support Center for Japan Environment and Children’s Study, National Center for Child Health and Development, Tokyo, Japan; 3 Department of Nutrition and Metabolic Medicine, Center for Preventive Medical Sciences, Chiba University, Chiba, Japan; 4 Department of Bioenvironmental Medicine, Graduate School of Medicine, Chiba University, Chiba, Japan

## Abstract

**Importance:**

It is unclear whether increased television (TV) and DVD viewing in early childhood from age 1 year decreases development or whether poor development increases TV/DVD viewing.

**Objective:**

To investigate the directional association between TV/DVD screen time and performance on developmental screeners in children aged 1 to 3 years.

**Design, Setting, and Participants:**

This longitudinal cohort study analyzed data from 57 980 children and mothers from a national birth cohort, the Japan Environment and Children’s Study. Data were collected in collaboration with 15 regional centers across Japan. The mothers were recruited between January 2011 and March 2014. Analyses using random intercept, cross-lagged panel models were performed for children aged 1, 2, and 3 years. Of 100 303 live births, children with missing developmental screening test scores and screen time data, those with congenital diseases or cerebral palsy, and those diagnosed with an autism spectrum disorder were excluded. Statistical analyses were conducted from October 2022 to July 2023.

**Exposures:**

TV and DVD screen time.

**Main Outcomes and Measures:**

Child development at ages 1, 2, and 3 years was assessed via the mother’s or guardian’s report using the Ages and Stages Questionnaire, third edition.

**Results:**

Of 57 980 included children, 29 418 (50.7%) were male, and the mean (SD) maternal age at delivery was 31.5 (4.9) years. A negative association between screen time and developmental scores was observed. Increased TV/DVD screen times at age 1 and 2 years were associated with lower developmental scores at age 2 and 3 years, respectively (2 years: β = −0.05; 95% CI, −0.06 to −0.04; 3 years: β = −0.08; 95% CI, −0.09 to −0.06). An obverse association was observed from the Ages and Stages Questionnaires, third edition, score in the communication domain at age 1 and 2 years to subsequent screen time (2 years: γ = −0.03; 95% CI, −0.04 to −0.02; 3 years: γ = −0.06; 95% CI, −0.07 to −0.04).

**Conclusions and Relevance:**

In this study, increased TV/DVD screen time from age 1 year negatively affected later development. To reduce the negative consequences of excessive media use, researchers and health care professionals should encourage family media management and recommend social support for parents who tend to rely on the media.

## Key Points

**Question:**

Is increased television/DVD screen time in infants associated with poor performance on developmental screeners?

**Findings:**

In this cohort study of 57 980 children, increased television/DVD screen time in children aged 1 and 2 years was associated with lower developmental scores at 2 and 3 years, respectively. Lower development scores were associated with increased screen time in children with maternal psychological distress.

**Meaning:**

In this study, increased screen time in early childhood was negatively associated with poor performance on developmental screeners, suggesting the need to support parents in creating family media plans.

## Teaser

This cohort study evaluates the directional association between television/DVD screen time and performance on developmental screeners in children aged 1 to 3 years.

## Introduction

Researchers have reported a significant increase in the number of children with neurodevelopmental and mental health problems, including language, learning, behavioral, and emotional disorders.^1,2^ This increase indicates the need to better understand the social, medical, and environmental factors underlying developmental problems in early childhood.

Screens, such as television (TV), are ubiquitous in the homes of young children. Although pediatric guidelines recommend that screen viewing be avoided for infants younger than 2 years and limited to 1 hour per day between the ages 2 and 5 years,^3,4,5^ many children fail to adhere to these recommendations.^6^ Excessive screen time in children younger than 3 years is associated with adverse effects on cognitive, language, motor skills, and social-behavioral development.^7,8,9,10,11^ Conversely, children with poor self-regulation are more likely to experience more exposure to screens.^12,13,14,15,16^ However, there is limited evidence for a causal relationship in early childhood regarding whether media exposure leads to developmental delays or whether children with developmental delays are more exposed to the media. After accounting for individual traitlike differences, several studies have used the random-intercepts, cross-lagged panel model (RI-CLPM) to clarify the bidirectional association between screen time and development (from age 2 to 3 years).^17,18^

Using the RI-CLPM and Ages and Stages Questionnaires (ASQ) as a developmental screener, Madigan et al^17^ showed that increased screen time at ages 2 and 3 years led to delayed achievement of developmental milestones at ages 3 and 5 years, respectively. The current study provides more convincing evidence of the role of screen use on child development and justifies the RI-CLPM used to analyze the bidirectional association. Although brain development during the first 3 years of life is critical, as it sets the foundation for future cognitive and emotional functioning,^19,20^ to our knowledge, no study has found a bidirectional relationship between screen time and development in young children, including those younger than 2 years. We replicated and extended the study of Madigan et al^17^ with a larger sample size, at a slightly early age, and with differential cultural backgrounds. Maternal prenatal or postpartum stress and child’s sex may be associated with cognitive functioning and behavioral problems in children.^21,22,23,24,25,26^ Detecting interactions is crucial for public health if maternal stress influences the fetal or children’s environment.

We aimed to examine the bidirectional association between TV/DVD screen time and developmental milestones during early childhood. Additionally, we examined the association in 5 specific domains and moderation or interaction effect by child’s sex and maternal distress. Children with psychiatric disorders, such as autism spectrum disorder (ASD), have been reported to have extreme screen time.^27,28^ Therefore, we analyzed data from the Japanese nationwide birth cohort study on 57 980 children aged 1, 2, and 3 years with primarily typical development, excluding those diagnosed with ASD.

## Methods

### Study Design and Participants

The participants included children from the Japan Environment and Children’s Study (JECS). The study design of JECS has been described previously.^29^ Briefly, 103 060 pregnancies were registered between January 2011 and March 2014 in 15 regional centers across Japan. We used the dataset jecs-ta-20190930. We included 100 303 live births, excluding 1254 miscarriages, 382 stillbirths, and 2123 with missing birth data. Next, children were excluded if they met any of the following criteria: missing developmental score data (n = 35 914) or missing TV/DVD screen time data (n = 794) at age 1, 2, or 3 years; had congenital diseases, cerebral palsy, or missing data (n = 5376); and diagnosed with ASD (eg, autism, pervasive developmental disorder, and Asperger syndrome) at age 3 years or missing data (n = 239). A total of 57 980 children were included in the analysis (eFigure in Supplement 1). Written informed consent was obtained from all the participants. The JECS protocol was reviewed and approved by the Ministry of the Environment’s Institutional Review Board on Epidemiological Studies and the ethics committees of all participating institutions. The study followed the Strengthening the Reporting of Observational Studies in Epidemiology (STROBE) reporting guideline.^30^

### Measures

#### Developmental Screener

Children’s development was assessed by the scores on the Japanese version of the ASQ, third edition (ASQ-3), completed by the parents or guardians of children aged 1, 2, and 3 years. The ASQ-3 is widely used for assessing development in communication, gross motor, fine motor, problem solving, and personal-social domains (eTable 1 in Supplement 1).^31^ Each domain comprises 6 questions, and their total scores range from 0 to 60 points per domain, with higher scores indicating better development. The Japanese version of ASQ-3 was validated with moderate to high sensitivity (75.3% to 100.0%) and specificity (66.7% to 100.0%).^32^ The mean ASQ-3 scores of 5 domains or each domain were used as continuous variables.

#### Screen Time

At ages 1, 2, and 3 years, parents or guardians were asked to report their children’s TV/DVD screen time on by asking, “How many hours do you let your children watch TV or DVD in a typical day?” The responses were collected from among 5 categories: none, less than 1 hour, 1 hour or more but less than 2 hours, 2 hours or more but less than 4 hours, and 4 hours or more. The categories were converted to corresponding numbers (0 hours, 0.5 hours, 1.5 hours, 3 hours, and 4.5 hours per day, respectively).

#### Predictors

In the model to explore the contribution of time-invariant factors on screen time and child development, possible predictors were selected a priori based on the literature,^17,27^ biological plausibility, and using a directed acyclic graph. *z* Scores for the following variables were included in the predictor model using the forced entry method: sex of the child, maternal age at delivery, maternal education, annual household income, maternal psychological distress assessed by the Kessler Psychological Distress Scale Japanese version^33^ at 1 year post partum, the presence of an elder sibling(s), attendance at a childcare facility, frequency of reading to the child, frequency of taking the child out of the house (other than the childcare facility), and child’s sleep time per day at age 1 year. Categorization is illustrated in eTable 2 in Supplement 1. Data were obtained from the questionnaire completed by the children’s parents or guardians as well as medical record transcripts.

### Statistical Analysis

Descriptive statistics were used to summarize the distribution of participant characteristics. We examined the longitudinal and directional associations between TV/DVD screen time and ASQ-3 scores using a RI-CLPM (Figure; eAppendix in Supplement 1). This model is a structural equation-based approach to better approximate within-person (time-variant) relationships by accounting for between-person (time-invariant, traitlike) stability by including random intercepts to the standard cross-lagged panel model.^34^ Owing to the nonnormal distribution of screen time and ASQ-3 scores, we used an asymptotic distribution-free estimation method.

**Figure.**
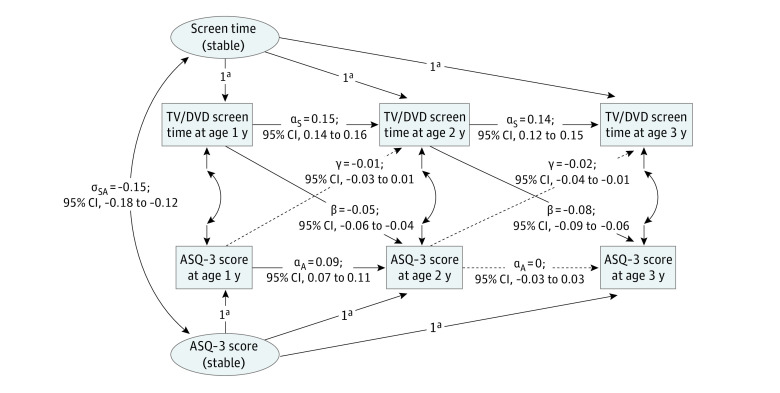
Random-Intercepts Cross-Lagged Panel Model for Associations Between Television (TV)/DVD Screen Time and Developmental Outcomes (Ages and Stages Questionnaires, Third Edition [ASQ-3], Scores) Standardized estimates with 95% CIs are presented. Solid lines in cross-lag and autoregressive links represent the absolute value of the standardized estimate, which is 0.03 or more. Dashed lines represent those less than 0.03. ^a^The factor loadings constrained to 1.

Cross-lag association of the screen time with ASQ-3 scores (β) and the association of ASQ-3 scores with screen time (γ) were estimated in 3 types of RI-CLPM. First, to identify associations between screen time and overall or domain-specific development, we performed estimation using the basic RI-CLPM (Figure) using mean ASQ-3 scores or scores of each domain, respectively. Second, to explore the interaction effects on the association between screen time and child development, we performed a multiple-group RI-CLPM stratified by the child’s sex and maternal psychological distress. To evaluate the interaction effect, we compared a model in which the lagged regression coefficients were constrained to be equal between the 2 groups with an unconstrained model, with differences in the fit indices between models. We considered the interaction effect to be insignificant when the difference in the comparative fit index (CFI) was less than 0.010, the change in the root mean square error of approximation (RMSEA) was less than 0.015, and the change in the standardized root mean square residual (SRMR) was less than 0.010.^35^ Third, to explore the contribution of predictors for screen time and child development, we constructed a predictor-included RI-CLPM by including between-level predictors affecting the random intercepts simultaneously, as presented by Mulder and Hamaker.^36^ In all models, benchmark values for interpreting the size of the RI-CLPM cross-lag effect were set at 0.03 (small effect), 0.07 (medium effect), and 0.12 (large effect).^37^

To assess the robustness of the findings, we performed sensitivity analyses using the basic RI-CLPM for children with complete data at 3 years (n = 70 226). In these analyses, we estimated missing TV/DVD screen time data and ASQ-3 scores at 1 and 2 years using full information maximum likelihood estimation and Bayesian estimation with the Markov chain Monte Carlo algorithm. The number of cohort participants included and excluded from the analyses and a comparison of basic demographic characteristics are summarized in eTable 3 in Supplement 1. All statistical analyses were conducted using SPSS and SPSS Amos version 27 (IBM).

## Results

### Descriptive Statistics

eTable 2 in Supplement 1 summarizes the characteristics of children and mothers. Of 57 980 included children, 29 418 (50.7%) were male, and the mean (SD) maternal age at delivery was 31.5 (4.9) years. At ages 1, 2, and 3 years, 15 051 children (26.0%), 16 430 children (28.3%), and 17 403 children (30.0%) viewed TV/DVD for at least 2 hours per day, respectively.

### Directional Association Between Screen Time and ASQ-3 Scores

The Figure and eTable 4 in Supplement 1 summarize the estimates in the basic RI-CLPM. Fit indices demonstrated good model fit. Significant variances of random intercepts were observed for both TV/DVD screen time (σ_S_^2^ = 0.46; 95% CI, 0.44-0.48) and ASQ-3 scores (σ_A_^2^ = 33.95; 95% CI, 32.31-35.59). This finding indicated stable individual differences in both screen time and developmental scores; furthermore, it was reasonable to set a random intercept in the model. Positive autoregressive associations in the screen time (α_S2_ and α_S3_) implied that children with more TV screen time were more likely to continue this behavior on subsequent occasions. We observed negative covariances between random intercepts of TV/DVD screen time and ASQ-3 scores (σ_SA_ = −0.15; 95% CI, −0.18 to −0.12), suggesting that children with more screen time tended to have lower ASQ-3 scores at ages 1 to 3 years.

Furthermore, we observed small to medium negative cross-lags linking from screen time at age 1 year to ASQ-3 score at age 2 years (β = −0.05; 95% CI, −0.06 to −0.04) and medium linking from screen time at age 2 years to ASQ-3 score at age 3 years (β = −0.08; 95% CI, −0.09 to −0.06).

### Association Between Screen Time and ASQ-3 Scores in Each Domain

Each model demonstrated a good fit (Table 1). We observed small to medium linking from screen time at age 1 year to ASQ-3 scores at age 2 years in the communication domain (β = −0.06; 95% CI, −0.07 to −0.05) and small to large linking from screen time at age 2 years to ASQ-3 scores at age 3 years in gross motor, fine motor, and personal-social domains (β range, −0.03 [95% CI, −0.05 to −0.01] to −0.12 [95% CI, −0.14 to −0.11]). The small to medium obverse linking was observed from ASQ-3 scores in the communication domain to screen time (2 years: γ = −0.03; 95% CI, −0.04 to −0.02; 3 years: γ = −0.06; 95% CI, −0.07 to −0.04).

**Table 1.** Directional Association Between Television/DVD Screen Time and Development (Ages and Stages Questionnaires, Third Edition [ASQ-3] Scores) in Each Domain

Association	Standardized estimate (95% CI)^a^				
	Communication	Gross motor	Fine motor	Problem-solving	Personal-social
Cross-lagged effects					
Screen time at age 1 y and ASQ-3 score at age 2 y, β	−0.06 (−0.07 to −0.05)	−0.01 (−0.02 to 0)	−0.01 (−0.03 to 0)	−0.01 (−0.02 to 0)	−0.01 (−0.03 to 0)
ASQ-3 score at age 1 y and screen time at age 2 y, γ	−0.03 (−0.04 to −0.02)	0.01 (0 to 0.02)	0 (−0.01 to 0)	−0.01 (−0.02 to 0)	0 (−0.01 to 0.01)
Screen time at age 2 y and ASQ-3 score at age 3 y, β	−0.02 (−0.04 to −0.01)	−0.03 (−0.05 to −0.01)	−0.07 (−0.08 to −0.06)	−0.01 (−0.03 to 0)	−0.12 (−0.14 to −0.11)
ASQ-3 score at age 2 y and screen time at age 3 y, γ	−0.06 (−0.07 to −0.04)	0.02 (0.01 to 0.03)	−0.02 (−0.04 to −0.01)	0.02 (0 to 0.03)	−0.01 (−0.02 to 0.01)
Fit indices					
CFI	1.00	1.00	1.00	1.00	1.00
RMSEA (90% CI)	0.01 (0.01 to 0.02)	0.02 (0.01 to 0.03)	0.03 (0.02 to 0.03)	0.02 (0.01 to 0.02)	0 (0 to 0.01)
SRMR	0	0.01	0.01	0	0

Abbreviations: CFI, comparative fit index; RI-CLPM, random-intercepts, cross-lagged panel model; RMSEA, root mean square error of approximation; SRMR, standardized root mean square residual.

^a^ Benchmark values for interpreting the size of the RI-CLPM cross-lag effect were set at 0.03 (small effect), 0.07 (medium effect), and 0.12 (large effect).^37^

### Moderation or Interaction Effects on the Cross-Lags Link

Table 2 and Table 3 present the results of 2 multiple-group RI-CLPMs stratified by the child’s sex and maternal psychological distress. Both models demonstrated a good fit. In the model stratified by the child’s sex, we observed no difference in the cross-lag effect between boys and girls (Table 2). For the model stratified by maternal psychological distress, we observed no distinct difference between the groups as assessed by a change in fit indices (change in CFI, 0; change in RMSEA, 0; change in SRMR, 0). However, a small to medium cross-lag association between ASQ scores at age 2 years and screen time at age 3 years was observed only in children with maternal psychological distress (γ = −0.05; 95% CI, −0.07 to −0.02) (Table 3).

**Table 2.** Directional Association Between Television/DVD Screen Time and Development (Ages and Stages Questionnaires, Third Edition [ASQ-3] Scores) Stratified By Child’s Sex

Association	Standardized estimate (95% CI)^a^	
	Boys	Girls
Cross-lagged effects		
Screen time at age 1 y and ASQ-3 score at age 2 y, β	−0.05 (−0.07 to −0.04)	−0.03 (−0.05 to −0.02)
ASQ-3 score at age 1 y and screen time at age 2 y, γ	−0.01 (−0.03 to 0.01)	0 (−0.01 to 0.01)
Screen time at age 2 y and ASQ-3 score at age 3 y, β	−0.09 (−0.11 to −0.07)	−0.07 (−0.09 to −0.05)
ASQ-3 score at age 2 y and screen time at age 3 y, γ	−0.02 (−0.04 to 0.01)	−0.01 (−0.03 to 0.02)
Fit indices		
CFI	1.00	
RMSEA (90% CI)	0.01 (0.01 to 0.02)	
SRMR	0	

Abbreviations: CFI, comparative fit index; RI-CLPM, random-intercepts, cross-lagged panel model; RMSEA, root mean square error of approximation; SRMR, standardized root mean square residual.

^a^ Benchmark values for interpreting the size of the RI-CLPM cross-lag effect were set at 0.03 (small effect), 0.07 (medium effect), and 0.12 (large effect).^37^

**Table 3.** Directional Association Between Television/DVD Screen Time and Development (Ages and Stages Questionnaires, Third Edition [ASQ-3] Scores) Stratified By Maternal Psychological Distress

Association	Standardized estimate (95% CI)^a^	
	Mentally stable (<5)^b^	Mental distress (≥5)^b^
Cross-lagged effects		
Screen time at age 1 y and ASQ-3 score at age 2 y, β	−0.04 (−0.05 to −0.03)	−0.06 (−0.09 to −0.04)
ASQ-3 score at age 1 y and screen time at age 2 y, γ	−0.01 (−0.03 to 0.01)	−0.02 (−0.04 to 0)
Screen time at age 2 y and ASQ-3 score at age 3 y, β	−0.08 (−0.09 to −0.06)	−0.09 (−0.12 to −0.06)
ASQ-3 score at age 2 y and screen time at age 3 y, γ	−0.02 (−0.03 to −0.01)	−0.05 (−0.07 to −0.02)
Fit indices		
CFI	1.00	
RMSEA (90% CI)	0.01 (0 to 0.01)	
SRMR	0	

Abbreviations: CFI, comparative fit index; NA, not applicable; RI-CLPM, random-intercepts, cross-lagged panel model; RMSEA, root mean square error of approximation; SRMR, standardized root mean square residual.

^a^ Benchmark values for interpreting the size of the RI-CLPM cross-lag effect were set at 0.03 (small effect), 0.07 (medium effect), and 0.12 (large effect).^37^

^b^ Maternal psychological distress was assessed by the Kessler Psychological Distress Scale Japanese version^33^ at 1 year post partum.

### Predictors of Screen Time and ASQ-3 Scores

The RI-CLPM with 10 predictors demonstrated a close model fit, although poorer than the basic RI-CLPM (CFI, 0.94; RMSEA, 0.04; 90% CI, 0.04-0.04; SRMR, 0.02) (data not shown). Increased TV/DVD screen time was associated with not attending a childcare facility, no elder sibling(s), infrequent reading to children, lower maternal education level, psychological distress, lower income, seldom going outdoors, less sleep time, and younger maternal age (Table 4). In contrast, higher developmental performance was associated with being female, frequent reading to the child, attending a childcare facility, elder sibling(s), younger maternal age, no psychological distress, frequently going outdoors, higher income, and more sleep time. Even after accounting for the effects of these predictors, the association between random intercepts of screen time and ASQ-3 remained negative (σ_SA_ = −0.11; 95% CI, −0.14 to −0.08) (data not shown).

**Table 4.** Predictors of Television/DVD Screen Time and Child Development Among 53 085 Children

Predictor	Standardized estimate (95% CI)	
	Screen time	Development (ASQ-3 score)
Maternal age	−0.03 (−0.04 to −0.02)	−0.11 (−0.12 to −0.10)
Maternal education	−0.11 (−0.12 to −0.10)	0.02 (0.01 to 0.03)
Household income (≥¥4 million [≥US $27 863] vs <¥4 million [<US $27 863])	−0.05 (−0.06 to −0.04)	0.04 (0.03 to 0.05)
Maternal psychological distress (yes vs no)^a^	0.05 (0.04 to 0.06)	−0.09 (−0.10 to −0.08)
Elder sibling (yes vs no)	−0.19 (−0.20 to −0.18)	0.14 (0.13 to 0.15)
Attending childcare facility (yes vs no)	−0.28 (−0.29 to −0.27)	0.16 (0.15 to 0.17)
Reading to child	−0.13 (−0.14 to −0.11)	0.18 (0.17 to 0.19)
Going outdoors	−0.04 (−0.05 to −0.03)	0.07 (0.06 to 0.08)
Sleep time	−0.03 (−0.04 to −0.02)	0.05 (0.04 to 0.05)
Child’s sex (girls vs boys)	0 (−0.01 to 0.02)	0.24 (0.23 to 0.25)
*R*^2^ for the random intercept	0.14	0.15

Abbreviation: ASQ-3, Ages and Stages Questionnaires, third edition.

^a^ Maternal psychological distress was assessed by the Kessler Psychological Distress Scale Japanese version^33^ at 1 year post partum. Participants were considered to be distressed if their score was 5 or greater.

### Sensitivity Analyses

The basic RI-CLPM was performed using full information maximum likelihood and Bayesian estimation for children with complete data only at age 3 years (n = 70 226). The results were similar to the RI-CLPM results for children with complete data at ages 1, 2, and 3 years (n = 57 980) (eTable 5 in Supplement 1).

## Discussion

Our study demonstrated that the negative cross-lag association from screen time to developmental scores remained consistent throughout toddlerhood. Notably, we found a bidirectional association between TV/DVD screen time and developmental scores in the communication domain from age 1 to 2 years. Additionally, we observed negative associations between TV/DVD screen time at age 2 years and the developmental scores in gross motor, fine motor, and personal-social domains at age 3 years. A negative association between the developmental score at age 2 years and screen time at age 3 years was observed in the communication domain. No significant interaction by child’s sex or maternal psychological distress was observed. In addition, developmental scores at age 2 years were negatively linked to screen time at age 3 years in children with maternal psychological distress.

Compared with another Japanese cohort that began in 2001,^38^ TV screen time for young children in the JECS cohort born between 2011 and 2014 appeared to decline. This finding was consistent with the decrease in TV and DVD screen times at age 0 to 8 years from 2011 to 2017 and is likely related to increased use of mobile devices.^39^ For TV/DVD screen time alone, only 25% to 29% of the children at ages 2 and 3 years met the guideline limiting screen time to no more than 1 hour each day.

Our study results support the findings of a Canadian cohort study using the RI-CLPM that demonstrated a directional association of greater screen time (TVs, DVDs, computers, and game consoles) at age 24 months with a lower ASQ-3 score at age 36 months.^17^ In addition, we found that screen time at age 1 year affects poorer communication skills at age 2 years. The effects on cognitive function may be partially mediated by reduced activity in the frontal and parietal brain regions.^40^ Conversely, screen time at age 2 years affected subsequent motor and personal-social skills. This may be attributed to missing opportunities to learn motor and interpersonal skills by sedentary looking at screens.

Our study demonstrated an obverse association from lower ASQ-3 scores only in the communication domain at age 1 year to more screen time at age 2 years and a similar association with a stronger effect from age 2 years to 3 years. The factors that may contribute to increased screen time include low development of communication skills, an environment that does not promote communication development, and a child’s preference for the environment. The association between lower ASQ-3 scores and subsequent increases in screen time was particularly evident among mothers experiencing mental health challenges. These findings underscore the importance of social support for mothers who may rely on media to manage their child’s behavior.

Frequent reading to the child, attending childcare, and having an elder sibling(s) were particularly associated with higher developmental scores and lesser screen time. A similar, albeit weaker, association was observed for taking children outdoors. This finding supports the existing literature^41,42,43,44,45,46,47,48^ by suggesting that parent-child verbal interaction, communication with others, and social play are effective in promoting development for young children, besides being associated with lesser screen time.

This study highlights several practical recommendations. First, emphasis should be placed on having opportunities for face-to-face interaction with family members and other children rather than relying on screens from an early age to enhance children’s development. Second, pediatricians and health care professionals should help each family develop a feasible media plan by making recommendations and introducing social support tools because parental attitudes toward media use significantly effect the home media environment.^49^ Third, promoting social networks that allow children to have high-quality interactions with their families is important. Active social interaction in the neighborhood may promote healthy behaviors, such as reducing screen time and increasing physical activity levels.^50^

### Strengths and Limitations

The strength of this study is that it provides insights into the directional association between TV/DVD screen time and developmental milestones in typically developing young children. It was based on a large birth cohort dataset representative of the Japanese population and provides reliable results.^51^ However, it had some limitations. First, we assessed the developmental milestones and screen time based on parental reports, which is suitable for collecting data from large populations; however, it may have led to reporting bias. Second, there is a single-informant bias, ie, most of the respondents to the questionnaire were mothers because they were the primary caregivers of their children. These biases may weaken the generalizability of the findings. Third, advances in media technology may have changed the situation of children’s screen viewing because children are increasingly exposed to portable media screens other than TVs and DVDs from an early age. According to a 2016 Japanese survey, smartphone, tablet, and portable gaming device use among children aged 1 to 3 years ranged from 2% to 32%.^52^ In a 2017 US survey, children aged 2 to 4 years spent 1 hour a day on both TV and mobile devices.^39^ Information on mobile device usage from age 1 year was not available for this study, which may underestimate total screen time. Fourth, the media content available to children is becoming increasingly diverse, and they may be exposed to content intended for adults. Further research is warranted on the effects of various screens and different types of content on children’s development.

## Conclusions

In this study, TV/DVD screen time was associated with lower subsequent developmental performance in young children. Screen media is prevalent in children’s environments, thus necessitating tailored media plans for each family for the better development of their child. In addition, parents experiencing emotional distress require support in reducing their children’s exposure to screens from an early age.

## Notes

Supplement 1. **eAppendix.** Construction of the Basic RI-CLPM Using Amos **eTable 1.** Description of ASQ-3 Domains **eTable 2.** Characteristics of Children and Mothers **eTable 3.** Comparison of Basic Demographic Characteristics of Children and Mothers **eTable 4.** Directional Association Between TV/DVD Screen Time (Hours/Day) and Development (ASQ-3 Scores) by the Basic RI-CLPM **eTable 5.** Directional Association Between TV/DVD Screen Time (Hours/Day) and Development (ASQ-3 Scores) by the Basic RI-CLPM Estimated By the Maximum Likelihood or Bayesian Estimation (N = 70 226) **eFigure.** Flowchart of the Inclusion Process of the Children

Supplement 2. Group Information. Japan Environment and Children’s Study Group

Supplement 3. Data Sharing Statement
