# Supplementary material for: Screen Time and Developmental Performance Among Children at 1-3 Years of Age in the Japan Environment and Children’s Study
Source: JAMA Pediatr. 2023 Sep 18;177(11):1168–75. doi: 10.1001/jamapediatrics.2023.3643 (PMC10507594; doi:10.1001/jamapediatrics.2023.3643)
Supplement: Supplement 1. — eAppendix. Construction of the Basic RI-CLPM Using Amos eTable 1. Description of ASQ-3 Domains eTable 2. Characteristics of Children and Mothers eTable 3. Comparison of Basic Demographic Characteristics of Children and Mothers eTable 4. Directional Association Between TV/DVD Screen Time (Hours/Day) and Development (ASQ-3 Scores) by the Basic RI-CLPM eTable 5. Directional Association Between TV/DVD Screen Time (Hours/Day) and Development (ASQ-3 Scores) by the Basic RI-CLPM Estimated By the Maximum Likelihood or Bayesian Estimation (N = 70 226) eFigure. Flowchart of the Inclusion Process of the Children [file jamapediatr-e233643-s001.pdf]

Supplemental Online Content

Yamamoto M, Mezawa H, Sakurai K, Mori C; Japan Environment and Children's Study Group. Screen time and developmental performance among children at 1-3 years of age in the Japan Environment and Children's Study. *JAMA Pediatr*. Published online September 18, 2023. doi:10.1001/jamapediatrics.2023.3643

eAppendix. Construction of the Basic RI-CLPM Using Amos
eTable 1. Description of ASQ-3 Domains
eTable 2. Characteristics of Children and Mothers
eTable 3. Comparison of Basic Demographic Characteristics of Children and Mothers
eTable 4. Directional Association Between TV/DVD Screen Time (Hours/Day) and Development (ASQ-3 Scores) by the Basic RI-CLPM
eTable 5. Directional Association Between TV/DVD Screen Time (Hours/Day) and Development (ASQ-3 Scores) by the Basic RI-CLPM Estimated By the Maximum Likelihood or Bayesian Estimation (N = 70 226)
eFigure. Flowchart of the Inclusion Process of the Children

This supplementary material has been provided by the authors to give readers additional information about their work.

© 2023 Yamamoto M et al. *JAMA Pediatrics*.

eAppendix. Construction of the Basic RI-CLPM Using Amos

The model is illustrated in Figure 1.

Firstly, observed variables were used to set within-person components for screen time and ASQ-3 scores at ages 1, 2, and 3 years. The structural relations were defined as follows:

1) Cross-lagged links (β_t and γ_t) were established to represent the longitudinal and directional associations between screen time and the ASQ-3 scores within individuals.

2) Autoregressive links (α_{St} and α_{At}) were established to capture within-person stability over time for both screen time and ASQ-3 scores.

Secondly, two latent variables were introduced to account for stable differences between individuals: B-Screen for screen time and B-ASQ for ASQ. Factor loadings toward within-person components of screen time and ASQ-3 at each time period were fixed at 1.

Thirdly, covariances were set for the random intercepts (σ_{SA}) and the within components of screen time and ASQ-3 at each time point in the model. Within the components, the residuals were allowed to covary.

© 2023 Yamamoto M et al. *JAMA Pediatrics*.

eTable 1. Description of ASQ-3 Domains

Domains	Focus
Communication	language skills, listening, and understanding
Gross Motor	movements using arms, legs, and other large muscles
Fine Motor	movements using hands and fingers
Problem Solving	playing with toys and learning
Personal-Social	solitary social play and interactions with others

[a]Abbreviation: Ages and Stages Questionnaires, third edition (ASQ-3)

© 2023 Yamamoto M et al. *JAMA Pediatrics*.

eTable 2. Characteristics of Children and Mothers

	N	(%)	Mean	(SD)
Number	57,980			
Sex of the child				
Male	29,418	(50.7)		
Maternal age at delivery, years			31.5	(4.9)
Missing	1	(0.0)		
Mother's education				
Junior or senior high	19,039	(32.8)		
Junior college or vocational	24,760	(42.7)		
Undergraduate or above	13,765	(23.7)		
Missing	416	(0.7)		
Household income, million Japanese Yen				
< 4	20,830	(35.9)		
≥ 4	33,420	(57.6)		
Missing	3,730	(6.4)		
Elder sibling				
None	26,670	(46.0)		
≥ 1	31,305	(54.0)		
Missing	5	(0.0)		
Maternal psychological distress at 1 year (K6 score)				
Mentally stable (<5)	45,512	(78.5)		
Mental distress (≥ 5)	11,967	(20.6)		
Missing	501	(0.9)		
Attending a childcare facility at 1 year				
Yes	14,442	(24.9)		
No	43,375	(74.8)		
Missing	163	(0.3)		
Reading to the child at 1 year				
Seldom	5,818	(10.0)		
1-3 times/month	6,464	(11.1)		
1-2 times/week	15,713	(27.1)		
3-4 times/week	12,986	(22.4)		
≥ 5 times/week	16,858	(29.1)		

© 2023 Yamamoto M et al. *JAMA Pediatrics.*

Missing	141	(0.2)		
Going outside at 1 year				
Seldom	240	(0.4)		
1-3 times/month	1,748	(3.0)		
1-2 times/week	15,682	(27.0)		
3-4 times/week	16,278	(28.1)		
≥ 5 times/week	23,906	(41.2)		
Missing	126	(0.2)		
Sleep time at 1 year, hours per day			12.9	(1.5)
Missing	354	(0.6)		
Screen time of TV/DVD, hours per day				
At 1 year				
none	6,035	(10.4)		
<1	19,569	(33.8)		
1– <2	17,325	(29.9)		
2– <4	11,361	(19.6)		
≥ 4	3,690	(6.4)		
At 2 years				
none	1,123	(1.9)		
<1	15,707	(27.1)		
1– <2	24,720	(42.6)		
2– <4	13,696	(23.6)		
≥ 4	2,734	(4.7)		
At 3 years				
none	966	(1.7)		
<1	13,817	(23.8)		
1– <2	25,794	(44.5)		
2– <4	14,758	(25.5)		
≥ 4	2,645	(4.6)		
ASQ-3 score, mean (SD)				
At 1 year				
Mean			41.7	(10.2)
Communication			37.6	(13.3)
Gross motor			42.9	(17.4)
Fine motor			48.3	(11.3)

© 2023 Yamamoto M et al. *JAMA Pediatrics*.

Problem solving			42.4	(13.4)
Personal-social			37.1	(14.4)
At 2 years				
Mean			49.1	(7.1)
Communication			45.5	(15.3)
Gross motor			54.1	(8.5)
Fine motor			50.0	(7.1)
Problem solving			49.2	(10.3)
Personal-social			46.6	(7.9)
At 3 years				
Mean			52.3	(7.7)
Communication			53.5	(9.9)
Gross motor			55.7	(7.7)
Fine motor			49.5	(12.3)
Problem solving			52.1	(10.4)
Personal-social			50.7	(9.6)
Respondents to the questionnaire				
At 1 year				
Mother	57,772	(99.6)		
Father	130	(0.2)		
Others	10	(0.0)		
Missing	68	(0.1)		
At 2 years				
Mother	57,679	(99.5)		
Father	206	(0.4)		
Others	14	(0.0)		
Missing	81	(0.1)		
At 3 years				
Mother	57,590	(99.3)		
Father	266	(0.5)		
Others	19	(0.0)		
Missing	105	(0.2)		
Primary caregivers				
At 1 year				
Mother	56,851	(98.1)		

© 2023 Yamamoto M et al. *JAMA Pediatrics*.

Father	424	(0.7)
Others	671	(1.2)
Missing	34	(0.1)
At 2 years (multiple answers available)		
Mother	57,381	(99.0)
Father	20,192	(34.8)
Others	11,131	(19.2)
Missing	84	(0.1)
At 3 years (multiple answers available)		
Mother	57,537	(99.2)
Father	22,148	(38.2)
Others	11,116	(19.2)
Missing	45	(0.1)

Abbreviations: standard deviation (SD); the Ages and Stages Questionnaires, third edition (ASQ-3); television/digital versatile disc (TV/DVD)

© 2023 Yamamoto M et al. *JAMA Pediatrics*.

eTable 3. Comparison of Basic Demographic Characteristics of Children and Mothers

	Included (N=57,980)				Excluded live births (N=42,323)				Effect size	
	N	(%)	Mean	(SD)	N	(%)	Mean	(SD)	Phi or Cramer's V	Cohen's d
Sex of the child										
Male	29,418	(50.7)			21,978	(51.9)			0.01	
Maternal age at delivery, years										
Mean (SD)			31.5	(4.9)			30.7	(5.3)		0.15
Mother's education										
Junior or senior high	19,039	(32.8)			16,519	(39.0)			0.09	
Junior college or vocational	24,760	(42.7)			16,420	(38.8)				
Undergraduate or above	13,765	(23.7)			7,475	(17.7)				
Household income, million Japanese Yen										
< 4	20,830	(35.9)			15,947	(37.7)			0.04	
≥ 4	33,420	(57.6)			21,309	(50.3)				

[a]Abbreviation: standard deviation (SD)

© 2023 Yamamoto M et al. *JAMA Pediatrics*.

eTable 4. Directional Association Between TV/DVD Screen Time (Hours/Day) and Development (ASQ-3 Scores) by the Basic RI-CLPM

Association	Standardized estimate (95% CI)[a]	
Cross-lagged effects		
Screen time at age 1 y and ASQ-3 score at age 2 y, β	-0.05	(-0.06, -0.04)
ASQ-3 score at age 1 y and screen time at age 2 y, γ	-0.01	(-0.03, 0.01)
Screen time at age 2 y and ASQ-3 score at age 3 y, β	-0.08	(-0.09, -0.06)
ASQ-3 score at age 2 y and screen time at age 3 y, γ	-0.02	(-0.04, -0.01)
Autoregressive effects		
Screen time at age 1 y and age 2 y, α_S	0.15	(0.14, 0.16)
Screen time at age 2 y and age 3 y, α_S	0.14	(0.12, 0.15)
ASQ-3 score at age 1 y and age 2 y, α_A	0.09	(0.07, 0.11)
ASQ-3 score at age 2 y and age 3 y, α_A	0.00	(-0.03, 0.03)
Covariances (σ_{SA})		
B-Screen ↔ B-ASQ	-0.15	(-0.18, -0.12)
Variances		
B-Screen (σ_S^2)	0.46	(0.44, 0.48)
B-ASQ (σ_A^2)	33.95	(32.31, 35.59)
Fit indices		
CFI	1.00	
RMSEA (90% CI)	0.01	(0.01, 0.02)
SRMR	0.00	

Abbreviations: Ages and Stages Questionnaires, third edition (ASQ-3); comparative fit index (CFI); random-intercepts, cross-lagged panel model (RI-CLPM); root mean square error of approximation (RMSEA); and standardized root mean square residual (SRMR); television/digital versatile disc (TV/DVD)

[a]Benchmark values for interpreting the size of the RI-CLPM cross-lag effect were set at 0.03 9 (small effect), 0.07 (medium effect), and 0.12 (large effect).[37]

© 2023 Yamamoto M et al. *JAMA Pediatrics.*

eTable 5. Directional Association Between TV/DVD Screen Time (Hours/Day) and Development (ASQ-3 Scores) by the Basic RI-CLPM Estimated By the Maximum Likelihood or Bayesian Estimation (N = 70 226)

Association	Standardized estimate (95% CI)			
	Maximum likelihood		Bayesian	
Cross-lagged effects				
Screen time at age 1 y and ASQ-3 score at age 2 y, β	-0.04	(-0.05, -0.03)	-0.04	(-0.05, -0.03)
ASQ-3 score at age 1 y and screen time at age 2 y, γ	-0.01	(-0.02, 0.01)	-0.01	(-0.02, 0.00)
Screen time at age 2 y and ASQ-3 score at age 3 y, β	-0.07	(-0.08, -0.06)	-0.07	(-0.08, -0.06)
ASQ-3 score at age 2 y and screen time at age 3 y, γ	-0.02	(-0.04, -0.01)	-0.02	(-0.03, -0.00)
Autoregressive effects				
Screen time at age 1 y and age 2 y, α_S	0.15	(0.14, 0.16)	0.15	(0.14, 0.16)
Screen time at age 2 y and age 3 y, α_S	0.13	(0.12, 0.15)	0.13	(0.12, 0.14)
ASQ-3 score at age 1 y and age 2 y, α_A	0.10	(0.08, 0.11)	0.09	(0.08, 0.11)
ASQ-3 score at age 2 y and age 3 y, α_A	0.01	(-0.02, 0.03)	0.01	(-0.02, 0.03)
Covariances (σ_{SA}), unstandardized				
B-Screen ↔ B-ASQ	-0.16	(-0.19, -0.13)		
Variances				
B-Screen (σ_S^2)	0.42	(0.40, 0.43)	0.46	(0.45, 0.48)
B-ASQ (σ_A^2)	34.69	(33.20, 36.19)	34.74	(33.17, 36.23)
Fit indices				
CFI	1.00			
RMSEA (90% CI)	0.01	(0.01, 0.02)		

Abbreviations: Ages and Stages Questionnaires, third edition (ASQ-3); comparative fit index (CFI); random-intercepts, cross-lagged panel model (RI-CLPM); root mean square error of approximation (RMSEA); television/digital versatile disc (TV/DVD)

© 2023 Yamamoto M et al. *JAMA Pediatrics.*

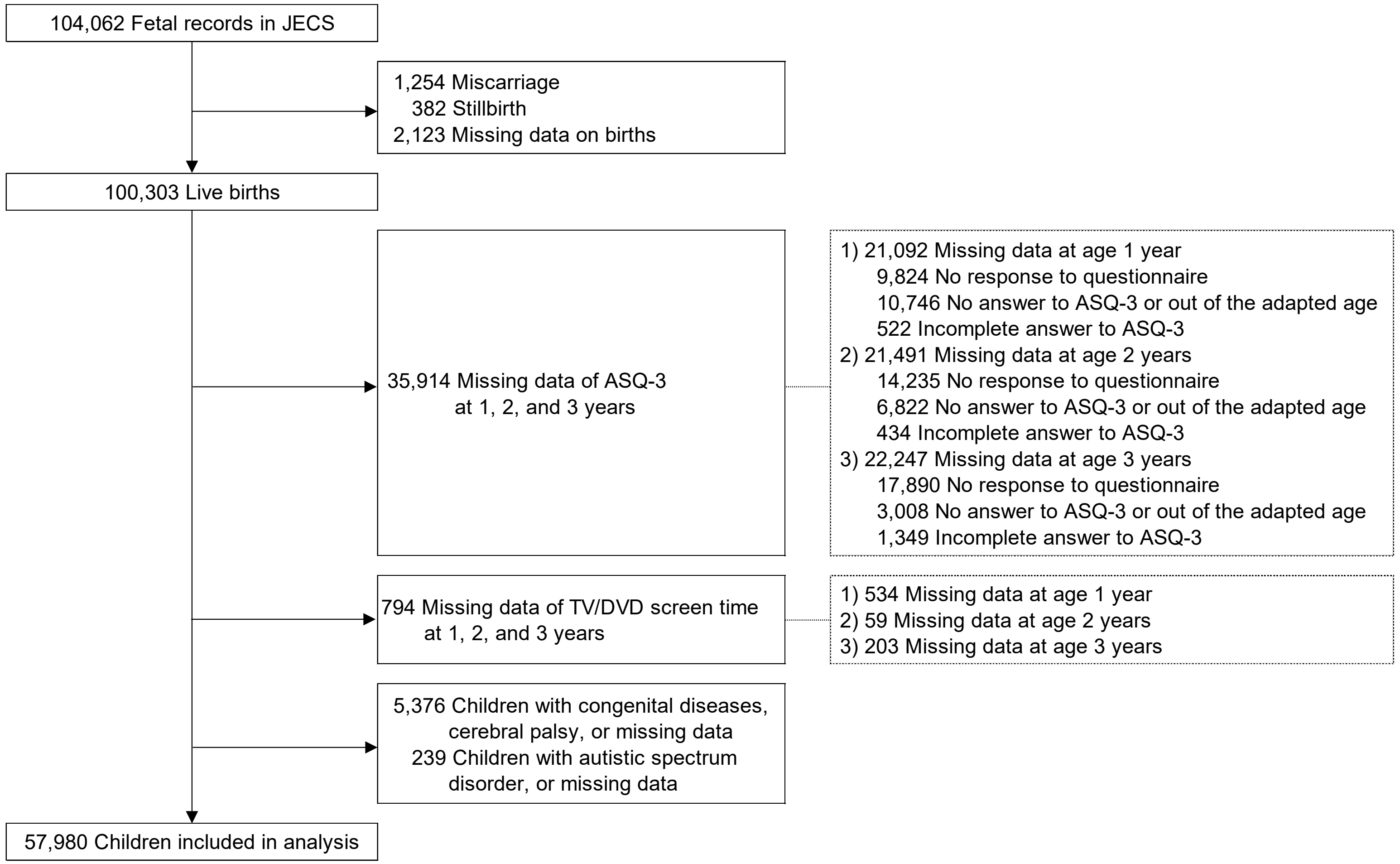

eFigure. Flowchart of the Inclusion Process of the Children

Abbreviations: Ages and Stages Questionnaires, third edition (ASQ-3); television/digital versatile disc (TV/DVD).

© 2023 Yamamoto M et al. *JAMA Pediatrics.*
