# Supplementary material for: Screen Time and Developmental Performance Among Children at 1-3 Years of Age in the Japan Environment and Children’s Study
Source: JAMA Pediatr. 2023 Sep 18;177(11):1168–75. doi: 10.1001/jamapediatrics.2023.3643 (PMC10507594; doi:10.1001/jamapediatrics.2023.3643)
Supplement: Supplement 3. — Data Sharing Statement [file jamapediatr-e233643-s003.pdf]

# Data Sharing Statement

Yamamoto. Screen Time and Developmental Performance Among Children at 1-3 Years of Age in the Japan Environment and Children's Study. *JAMA Pediatr*. Published September 18, 2023. doi:10.1001/jamapediatrics.2023.3643

**Data**
**Data available:** No

**Additional Information**
**Explanation for why data not available:** Data are unsuitable for public deposition due to ethical restrictions and the legal framework of Japan. It is prohibited by the Act on the Protection of Personal Information (Act No. 57 of May 30, 2003, amendment on September 09, 2015) to publicly deposit data containing personal information. Ethical Guidelines for Medical and Health Research Involving Human Subjects enforced by the Japan Ministry of Education, Culture, Sports, Science and Technology and the Ministry of Health, Labour and Welfare also restricts the open sharing of epidemiologic data. All inquiries about access to data should be sent to: jecs-en@nies.go.jp. The person responsible for handling inquiries sent to this e-mail address is Dr. Shoji F. Nakayama, JECS Programme Office, National Institute for Environmental Studies.
